# Apoptosis Induction of Human Bladder Cancer Cells by Sanguinarine through Reactive Oxygen Species-Mediated Up-Regulation of Early Growth Response Gene-1

**DOI:** 10.1371/journal.pone.0063425

**Published:** 2013-05-22

**Authors:** Min Ho Han, Cheol Park, Cheng-Yun Jin, Gi-Young Kim, Young-Chae Chang, Sung-Kwon Moon, Wun-Jae Kim, Yung Hyun Choi

**Affiliations:** 1 Anti-Aging Research Center & Blue-Bio Industry RIC, Dongeui University, Busan, Republic of Korea; 2 Department of Biochemistry, Dongeui University College of Oriental Medicine, Busan, Republic of Korea; 3 Department of Molecular Biology, College of Natural Sciences, Dongeui University, Busan, Republic of Korea; 4 School of Pharmaceutical Science, Zhengzhou University, Henan, China; 5 Laboratory of Immunobiology, Department of Marine Life Sciences, Jeju National University, Jeju, Republic of Korea; 6 Research Institute of Biomedical Engineering and Department of Medicine, Catholic University of Daegu School of Medicine, Daegu, Republic of Korea; 7 School of Food Science and Technology, Chung-Ang University, Ansung, Republic of Korea; 8 Department of Urology, Chungbuk National University College of Medicine, Cheongju, Republic of Korea; Henry Ford Health System, United States of America

## Abstract

Although the effects of sanguinarine, a benzophenanthridine alkaloid, on the inhibition of some kinds of cancer cell growth have been established, the underlying mechanisms are not completely understood. This study investigated possible mechanisms by which sanguinarine exerts its anticancer action in cultured human bladder cancer cell lines (T24, EJ, and 5637). Sanguinarine treatment resulted in concentration-response growth inhibition of the bladder cancer cells by inducing apoptosis. Sanguinarine-induced apoptosis was correlated with the up-regulation of Bax, the down-regulation of Bid and XIAP, the activation of caspases (-3, -8, and -9), and the generation of increased reactive oxygen species (ROS). The ROS scavenger N-acetyl cysteine (NAC) completely reversed the sanguinarine-triggered apoptotic events. In addition, sanguinarine effectively increased the activation of the c-Jun N-terminal kinase (JNK) and the expression of the early growth response gene-1 (Egr-1), which was recovered by pretreatment with NAC. Furthermore, knockdown of *Egr-1* expression by small interfering RNA attenuated sanguinarine-induced apoptosis, but not the JNK inhibitor, indicating that the interception of ROS generation blocked the sanguinarine-induced apoptotic effects via deregulation of the expression of Egr-1 proteins. Taken together, the data provide evidence that sanguinarine is a potent anticancer agent, which inhibits the growth of bladder cancer cells and induces their apoptosis through the generation of free radicals.

## Introduction

Benzo[c]phenanthridine alkaloids (BAs) are a relatively small group of isochinoline alkaloids, which have been detected in many plant species of the families Papaveraceae, Fumariaceae, Ranunculaceae, and Rutaceae [1]. Sanguinarine is a quaternary ammonium salt belonging to this group of BAs. It has been extracted from some plants, including bloodroot (*Sanguinaria canadensis* L.), the Mexican prickly poppy *Argemone mexicana* L., *Chelidonium majus,* and *Macleaya cordata*. [2], [3]. Sanguinarine has been shown to possess strong antibacterial and anti-inflammatory properties [4]–[6]. Recent data have also demonstrated that this compound can induce apoptosis in a variety of cancer cell lines *in vitro*; however, it does not show any toxic effects on normal cells when administered at similar doses [7]–[16].

Reactive oxygen species (ROS) are highly reactive molecules. They include superoxide anion radicals, hydrogen peroxide, singlet oxygen, and hydroxyl radicals. ROS are generally derived from the normal metabolism of oxygen, and mitochondria are the primary source of ROS. Although basal levels of ROS serve as a physiological regulator in normal cell proliferation and differentiation, high levels of ROS can cause severe damage to DNA and proteins, leading to apoptosis [17]–[19]. In addition, excessive oxidative stress particularly targets mitochondria, causing a loss of mitochondrial membrane potential (*ΔΨm*) and mitochondria-mediated apoptosis [17]–[19]. Recent studies suggest that the generation of ROS by sanguinarine initiates cascades of cell death signals in some human cancer cell lines *in vitro* [12], [13], [15].

Reactive oxygen species (ROS) are highly reactive molecules. They include superoxide anion radicals, hydrogen peroxide, singlet oxygen, and hydroxyl radicals. ROS are generally derived from the normal metabolism of oxygen, and mitochondria are the primary source of ROS. Although basal levels of ROS serve as a physiological regulator in normal cell proliferation and differentiation, studies have shown that high levels of ROS can cause severe damage to DNA and proteins, leading to apoptosis [17]–[19]. In addition, excessive oxidative stress particularly targets mitochondria, causing a loss of mitochondrial membrane potential (*ΔΨm*) and mitochondria-mediated apoptosis [17]–[19]. Recent studies have suggested that the generation of ROS by sanguinarine initiates cascades of cell death signals in some human cancer cell lines *in vitro* [12], [13], [15].

Among many redox-regulated genes, the early growth response-1 (Egr-1), a zinc-finger transcriptional factor, is of interest because it is rapidly and transiently induced by a number of extracellular stimuli [20]–[22] and by all inducers of ROS-mediated signaling and inflammation [23]–[28]. Therefore, Egr-1 can play a critical role in coordinating cellular events following oxidantive stress [29]–[31]. However, the role of Egr-1 in apoptosis signaling pathways activated by ROS in cancer cells treated with sanguinarine has not been delineated.

The present study used the human bladder cancer cell lines, T24, EJ, and 5637, to examine the cytotoxic efficacy of sanguinarine and to investigate the molecular mechanisms underlying the apoptotic activity caused by sanguinarine. The results showed that the sanguinarine-induced apoptotic signaling pathways modulated the activity of Bcl-2 and the inhibitor of apoptosis protein (IAP) family proteins and led to mitochondrial dysfunction, the activation of caspases, and the induction of Egr-1. The results also suggested that ROS are critical regulators of the sanguinarine-induced apoptotic events.

## Materials and Methods

### Cell Culture and Cell Viability Assay

Human bladder cancer cell lines (T24, EJ, and 5637) were obtained from the American Type Culture Collection (Rockville, MD, USA). The cells were cultured in RPMI 1640 medium, supplemented with 10% fetal bovine serum (FBS, Gibco-BRL, Gaithersburg, MD, USA) and 1% penicillin-streptomycin at 37°C in a humid environment containing 5% CO_2_. Sanguinarine (Sigma-Aldrich Chemical Co., St. Louis, MO, USA) was dissolved in methanol as a stock solution at a 10 mM concentration and was stored in aliquots at −20°C. For the cell viability study, cells were seeded in 6-well plates at a density of 1×10^5^ cells per well. After 24 h stabilization, the cells were treated with various concentrations of sanguinarine for a further 24 h. Following treatment, the viability of the cells was determined with the 3-(4,5-dimethylthiazol-2-yl)-2,5-diphenyltetrazolium bromide (MTT, Sigma-Aldrich) assay, which is based on the conversion of MTT to MTT formazan by mitochondrial enzymes.

### Nuclear Staining with DAPI

After treating the cells with sanguinarine for 24 h, they were harvested, washed in ice-cold phosphate-buffered saline (PBS), and fixed with 3.7% paraformaldehyde (Sigma-Aldrich) in PBS for 10 min at room temperature. The fixed cells were washed with PBS and stained with a 4,6-diamidino-2-phenylindole (DAPI, Sigma-Aldrich) solution (2.5 µg/ml) for 10 min at room temperature. Changes in the nuclear morphology of the cells were analyzed using a fluorescence microscope (Carl Zeiss, Germany).

### Flow Cytometry Analysis for the sub-G1 Phase

The cells were harvested and washed once with PBS, fixed in ice-cold 70% ethanol, and stored at 4°C. Prior to the analysis, the cells were washed once again with PBS, suspended in 1 ml of a cold propidium iodide (PI, Sigma-Aldrich) solution containing 100 mg/ml RNase A, 50 µg/ml PI, 0.1% (w/v) sodium citrate, and 0.1% (v/v) NP-40 and further incubated on ice for 30 min in the dark. Flow cytometric analyses were carried out using a flow cytometer (FACS Calibur; Becton Dickinson, San Jose, CA, USA), and Cell-Quest pro software was used to determine the relative DNA content based on the presence of red fluorescence [19].

### Detection of Apoptosis by Annexin-V FITC Staining

The cells were washed with PBS and resuspended in an annexin-V binding buffer containing 10 mM HEPES/NaOH (pH 7.4), 140 mM NaCl, and 2.5 mM CaCl_2_. Aliquots of the cells were incubated with annexin-V fluorescein isothiocyanate (FITC, R&D Systems; Minneapolis, MN, USA), mixed, and incubated for 15 min at room temperature in the dark. PI at a concentration of 5 µg/ml was added to distinguish the necrotic cells. The apoptotic cells (V^+^/PI^−^) were measured with a flow cytometer.

### Measurement of Intracellular ROS

ROS production was monitored using the stable nonpolar dye 2,7 dichlorofluorescein diacetate (DCFH-DA, Sigma-Aldrich). The cells were seeded in 24-well plates and incubated in the presence or absence of sanguinarine for different periods of time. Later, the cells were incubated with 10 mM DCFH-DA for 30 min. The ROS production in the cells was monitored with a flow cytometer using the Cell-Quest pro software [32]. The production of intracellular ROS was also monitored by the fluorescence emission of DCFH-DA within the cells using a fluorescent microscope.

### Protein Extraction and Western Blotting

The cells were harvested and washed twice in PBS at 4°C. Total cell lysates were lysed in a lysis buffer (40 mM Tris [pH 8.0], 120 mM, NaCl, 0.5% NP-40, 0.1 mM sodium orthovanadate, 2 µg/ml aprotinin, 2 µg/ml leupeptin, and 100 µg/ml phenymethylsulfonyl fluoride). The supernatants were collected, and the protein concentrations were measured using a Bio-Rad protein assay (Bio-Rad, Hercules, CA, USA) according to the manufacturer’s instructions. For the Western blot analysis, equal amounts of protein extracts were extracted from SDS-polyacrylamide gels and transferred to polyvinylidene difluoride membranes (Schleicher & Schuell, Keene, NH, USA) by electroblotting. The membranes were blocked with 5% nonfat dry milk in PBS with Tween 20 buffer (PBS-T) (20 mM Tris [pH 7.5], 100 mM NaCl, and 0.1% Tween 20) for 1 h at room temperature. The membranes were then incubated overnight at 4°C with the primary antibodies, probed with enzyme-linked secondary antibodies, and visualized by enhanced chemiluminescence (ECL; Amersham Corp, Arlington Heights, IL, USA) according to the recommended procedure. The primary antibodies were purchased from Santa Cruz Biotechnology Inc. (Santa Cruz, CA, USA) and Cell Signaling Technology Inc. (Boston, MA, USA). Peroxidase-labeled donkey antirabbit immunoglobulin and peroxidase-labeled sheep antimouse immunoglobulin were purchased from the Amersham Corp.

### In vitro Caspase Activity Assay

The caspase activities were determined by colorimetric assays using caspase-3, -8, and -9 activation kits according to the manufacturer’s protocol (R&D Systems). Briefly, the cells were lysed in a lysis buffer for 30 min in an ice bath. The supernatants were collected and incubated at 37°C with the reaction buffer, which contained dithiothreitol and the substrates Asp-Glu-Val-Asp (DEVD)-p-nitroaniline (pNA) for caspase-3, Ile-Glu-Thr-Asp (IETD)-pNA for caspase-8, and Leu-Glu-His-Asp (LEHD)-pNA for caspase-9. The optical density of the reaction mixture was quantified spectrophotometrically at a wavelength of 405 nm.

### Treatment with Small Interfering RNA

The cells were seeded in a 6-well plate at an initial density of 1.5×10^5^ cells per well. After 24 h of stabilization, they were transfected with 100 nM of small interfering RNA (siRNA) against human Egr-1 or an equal quantity of nonspecific irrelevant RNA (Dharmacon, Chicago, IL, USA) using a transfection reagent (Genefectine, Genetrone Biotech, Seoul, Korea), according to the manufacturer’s instructions. Following 24 h of transfection, the cells were incubated under the indicated conditions.

### Statistical Analysis

The data are expressed as means ± SD. Statistical comparisons were performed using SPSS 12.0 followed by Fisher’s test. Significant differences between the groups were determined using the unpaired Student’s *t*-test. A *p* value <0.05 was accepted as an indication of statistical significance.

## Results

### Effects of Sanguinarine on Cell Viability and Apoptosis Induction

**Figure 1:**
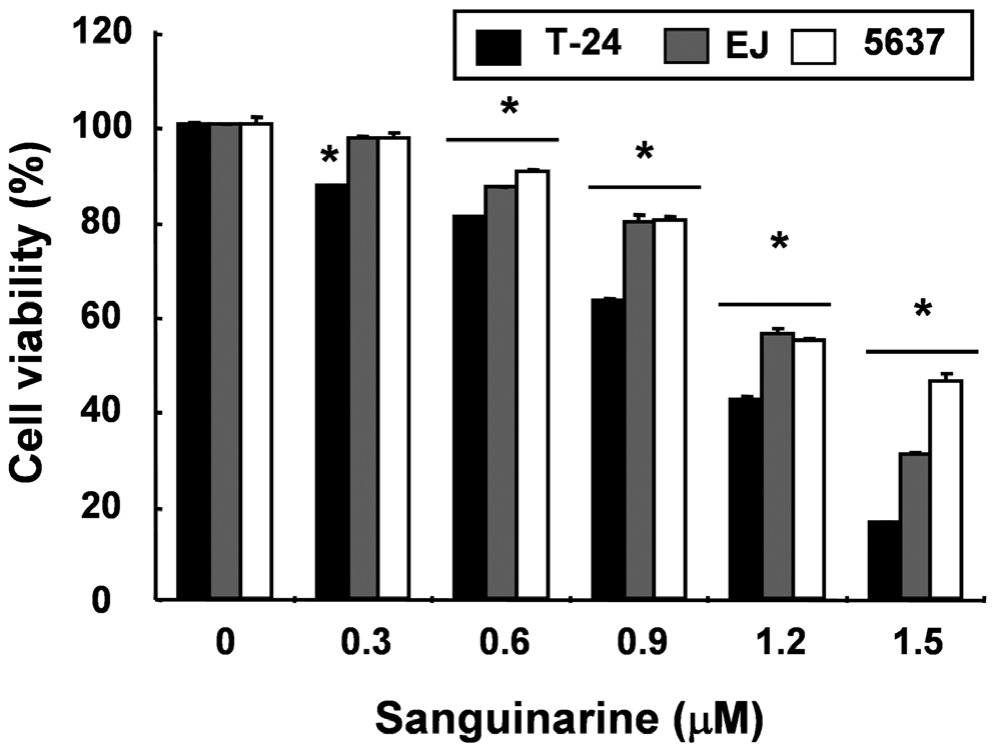
Inhibition of cell viability by sanguinarine in human bladder cancer cells. T24, 5637, and EJ cells were treated with the indicated concentrations of sanguinarine for 24 h, according to the measurements of cell viability with an MTT assay. Data are reported as means ± SD of three independent experiments. Significantly different from the control, *p<0.05.

**Figure 2:**
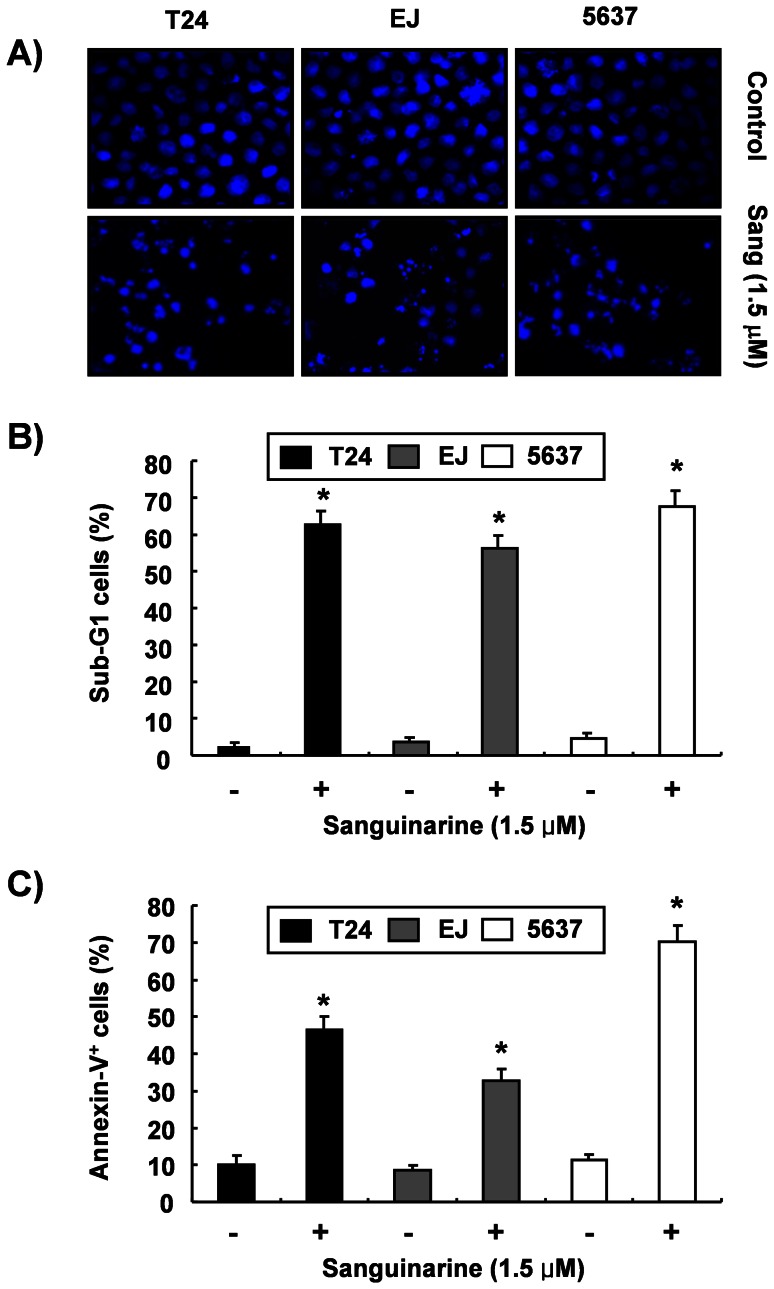
Induction of apoptosis by sanguinarine in the bladder cancer cells. (A) The cells were incubated with 1.5 µM sanguinarine for 24 h and then stained with DAPI. The stained nuclei were then observed under a fluorescent microscope (magnification, ×400) using a blue filter. (B) To quantify the degree of apoptosis induced by sanguinarine, the cells were evaluated for sub-G1 DNA content using a flow cytometer. (C) The cells were stained with FITC-conjugated annexin-V and PI for flow cytometry analysis. The apoptotic cells were determined by counting the percentage of annexin V(+), PI(−) cells and the percentage of annexin V(+), PI(+) cells. Data are reported as means ± SD of three independent experiments. Significantly different from the control, *p<0.05.

To investigate whether sanguinarine inhibited the proliferation of bladder cancer cells, three bladder cancer cell lines (T24, EJ, and 5637) were stimulated with the indicated concentrations of sanguinarine for 24 h, and an MTT assay was performed. As shown in Fig. 1, the treatment with sanguinarine decreased the viability of the bladder cancer cells in a concentration-dependent manner. Thus, further experiments were performed to determine whether this inhibitory effect of sanguinarine on the viability of the cells was the result of apoptotic cell death. First, DAPI staining determined morphological changes in the cells, as shown in Fig. 2A. Treatment with 1.5 µM sanguinarine resulted in a significant number of cells with chromatin condensation, loss of nuclear construction, and formation of apoptotic bodies, whereas these features were not observed in control cells. Second, flow cytometric analysis for the detection of hypodiploid cell populations determined the degrees of apoptosis in the cells treated with sanguinarine. As indicated in Fig. 2B, the addition of 1.5 µM sanguinarine to the bladder cells resulted in increased accumulations of cells in the sub-G1 phase. Third, flow cytometry analyses with annexin V and PI staining determined the magnitude of the apoptosis elicited by sanguinarine. As shown in Fig. 2C, the numbers of annexin V-positive cells showed marked increases in the sanguinarine-treated cells compared to the untreated control cells. Consequently, these data suggest that bladder cancer cells may undergo apoptosis after exposure to sanguinarine.

### Modulation of Bcl-2 and IAP Family Proteins, and Activation of Caspase by Sanguinarine

**Figure 3:**
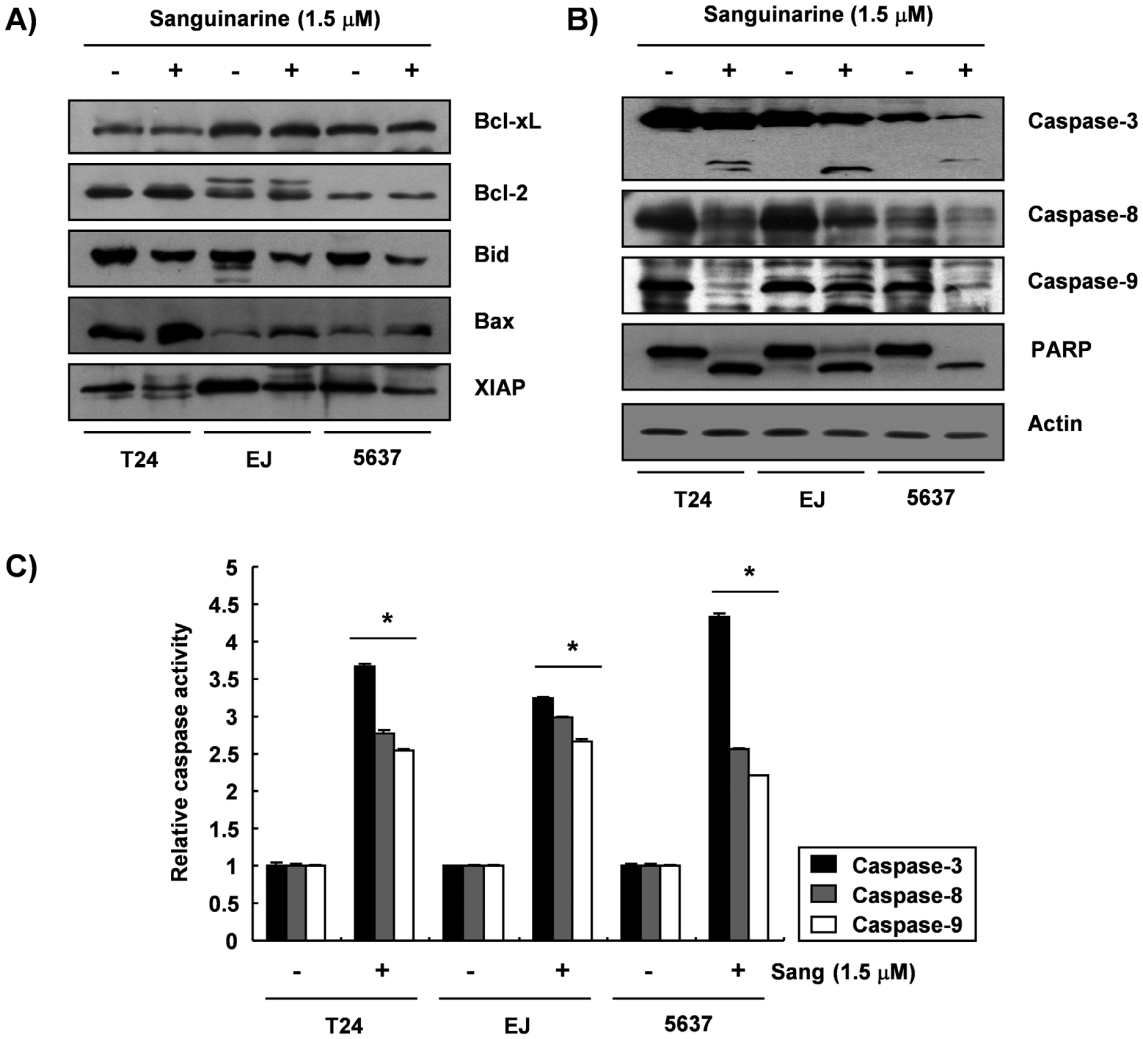
Effects of sanguinarine on the levels of the Bcl-2 family members, XIAP, and caspases and the activity of caspases in the bladder cancer cells. (A and B) After 24 h incubation with sanguinarine, the cellular proteins were separated by SDS-polyacrylamide gels and transferred onto nitrocellulose membranes. The membranes were probed with the indicated antibodies, and the proteins were visualized using an ECL detection system. To confirm equal loading, actin was used as an internal control. (C) To assay the *in vitro* caspase activity, aliquots were incubated with DEVD-pNA, IETD-pNA, and LEHD-pNA as substrates for caspase-3, -8, and -9, respectively, and then the released fluorescence products were measured. Each point represents the mean ± SD of the representative experiments performed at least three times. A Student’s *t*-test (*p<0.05 vs. untreated control) was used to analyze the statistical significance of the results.

The role of the Bcl-2 and the IAP family proteins was determined by Western blotting to investigate which mechanisms were involved in the sanguinarine-induced apoptosis in the bladder cancer cells. As shown in Fig. 3A, the treatment of the bladder cancer cells with 1.5 µM sanguinarine did not cause significant changes in the expression of the antiapoptotic proteins Bcl-2 and Bcl-xL. However, the levels of proapoptotic Bax increased and those of the antiapoptotic protein XIAP decreased in response to sanguinarine. In addition, the reduction in proapoptotic Bid proteins showed a marked increase with sanguinarine treatment in all the bladder cancer cell lines. To determine whether sanguinarine-induced apoptosis was associated with the activation of caspases, the expression and the activity of caspases in the sanguinarine-treated cells were examined. The results showed that the sanguinarine treatment down-regulated the levels of the procaspase-3 proteins and increased the levels of active-caspase-3. The levels of procaspase-8 and -9 proteins were also down-regulated in the sanguinarine-treated cells (Fig. 3B). For further quantification of the proteolytic activation of procaspase-3, -8, and -9, the lysates equalized by the protein from the cells treated with sanguinarine were assayed for their enzymatic activities. As shown in Fig. 3C, the sanguinarine treatment markedly increased their caspase activities. Subsequent Western blot analyses showed the progressive proteolytic cleavage of the poly (ADP-ribose) polymerase (PARP) protein, which is a downstream target of the activated caspase-3 [33], in the cells after the sanguinarine treatment (Fig. 3B).

### Sanguinarine-induced Apoptosis is Associated with the Generation of ROS

**Figure 4:**
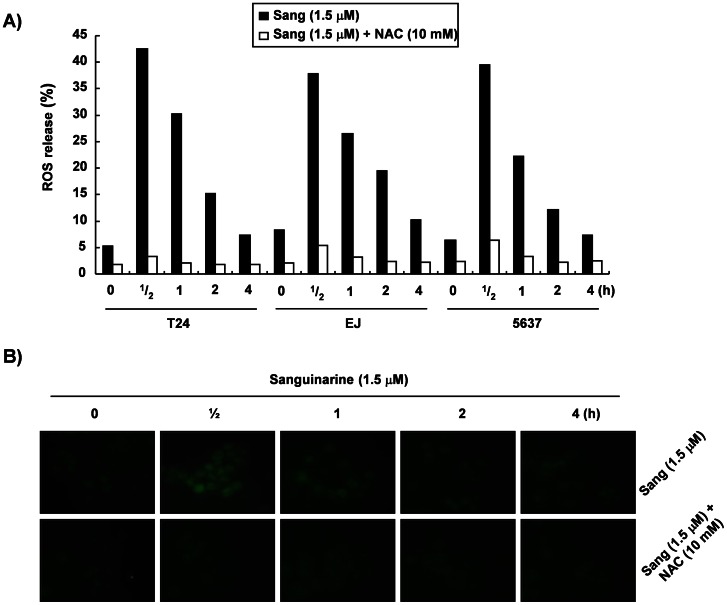
ROS generation by sanguinarine in the bladder cancer cells. (A) The cells were incubated with 1.5 µM sanguinarine for the indicated times, or they were pretreated with 10 mM NAC for 1 h and further treated with 1.5 µM sanguinarine for the indicated times. They were then stained with DCFH-DA. ROS generation was measured using a flow cytometer. Each point represents the mean of representative experiments performed twice. (B) DCFH fluorescence in T24 cells grown under the same conditions as (A) was determined under fluorescence using a green filter. At least five fields were viewed in each of the experiments.

**Figure 5:**
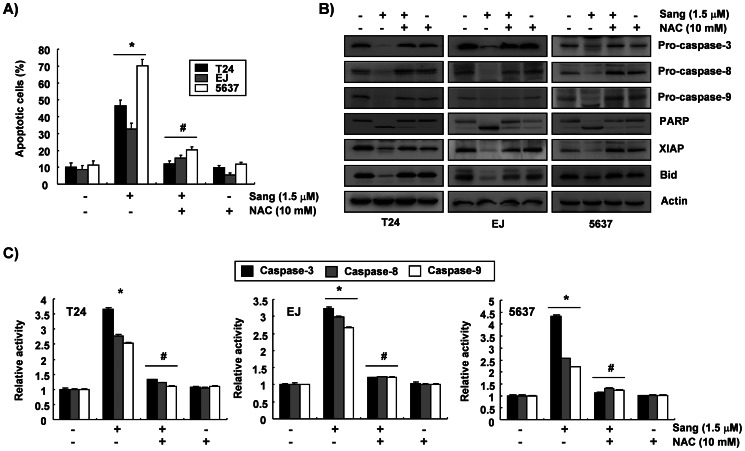
ROS-dependent apoptosis induction by sanguinarine in the bladder cancer cells. (A) The cells were treated with or without NAC (10 mM) for 1 h before challenge with 1.5 µM sanguinarine for 24 h. They were collected and stained with FITC-conjugated annexin-V and PI for flow cytometry analysis. Data are reported as means ± SD of three independent experiments. A Student’s *t*-test (*p<0.05 vs. untreated control; ^#^ *p*<0.05 vs. sanguinarine-treated cells) was used to analyze the statistical significance of the results. (B) They were then harvested, and the indicated proteins were detected by Western blot analysis. Actin was used as an internal control. (C) To assay the *in vitro* caspase activity, aliquots were incubated at 37°C for 1 h, and the released fluorescence products were measured. Each point represents the mean ± SD of representative experiments performed at least three times. A Student’s *t*-test (*p<0.05 vs. untreated control; ^#^ *p*<0.05 vs. sanguinarine-treated cells) was used to analyze the statistical significance of the results.

To determine whether sanguinarine-induced apoptosis was associated with ROS-mediated oxidative stress, intracellular ROS production was measured with the DCFH-DA fluorescence assay using a flow cytometer. As indicated in Fig. 4A, when the cells were exposed to sanguinarine, the level of intracellular ROS drastically increased at 30 min (more than an 8-fold increase compared to the control), and it decreased with time thereafter. Prior treatment of the cells with a well-known ROS scavenger, N-acetylcysteine (NAC), greatly diminished this heightened ROS level in the sanguinarine-treated cells. The production of intracellular ROS was also monitored by the fluorescence emission of DCFH-DA within T24 cells using a fluorescent microscope. The increased intensity of DCF-DA staining observed in the sanguinarine-treated cells was time-dependently abrogated to control levels in the presence of NAC (Fig. 4B). To determine whether sanguinarine-induced ROS production was attributable to the apoptosis induction, the cells were treated with NAC for 1 h and co-incubated with sanguinarine for a further 24 h. As shown in Fig. 5A, the inhibitory effects of NAC on sanguinarine-induced ROS production correlated with a marked inhibition of apoptotic cell death measured by the flow cytometer. Furthermore, blocking the generation of ROS by pretreating the cells with NAC prevented the sanguinarine-induced activation of caspases, the cleavage of PARP, and the modulation of Bcl-2 and IAP family proteins (Fig. 5B and C). Taken together, these data suggest that a ROS-generating system plays an essential role in sanguinarine-induced apoptosis in bladder cancer cells.

### Sanguinarine-induced Apoptosis is not Associated with the Activation of JNK

**Figure 6:**
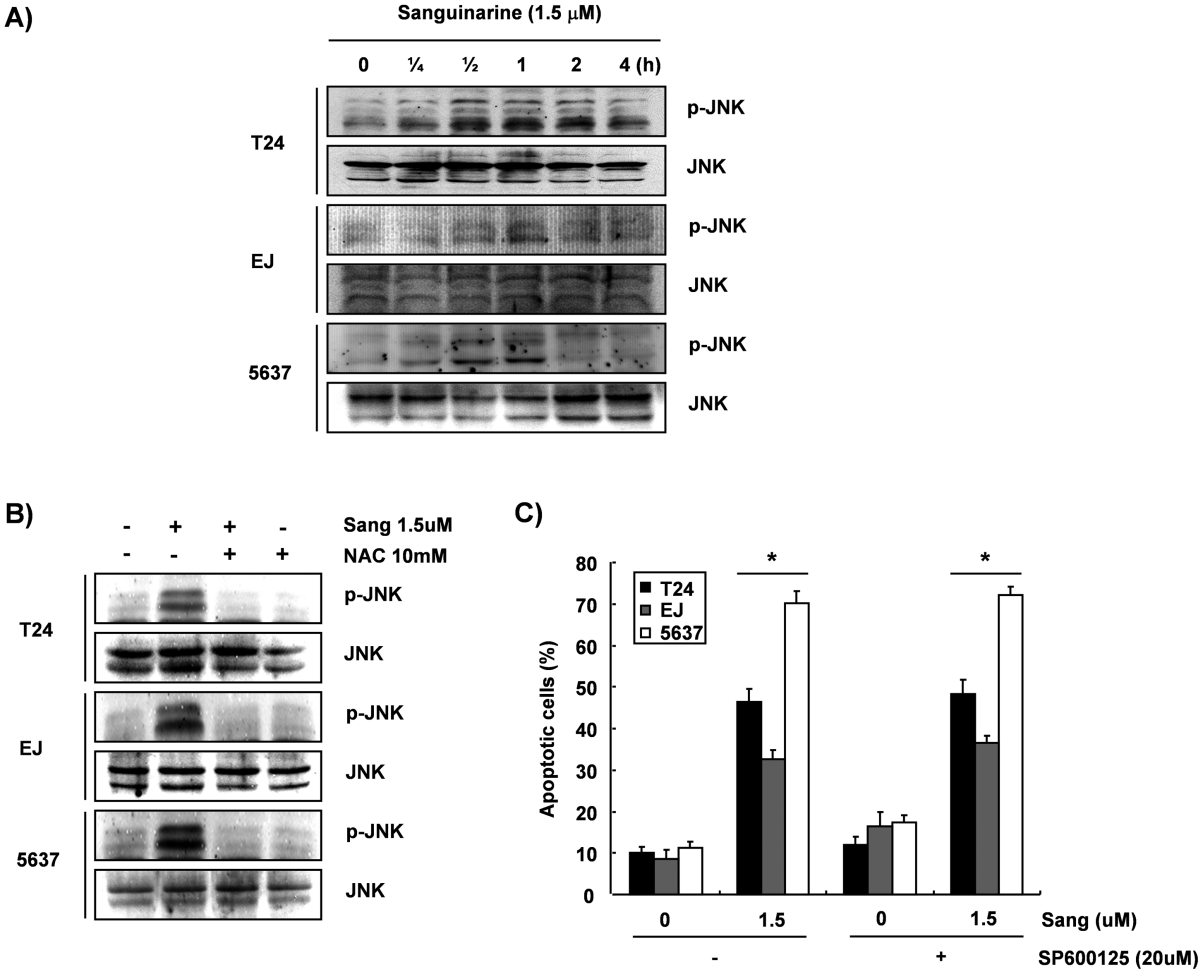
JNK activation in the bladder cancer cells in response to sanguinarine. (A) The cells were treated with sanguinarine for the indicated times, or (B) they were pretreated with NAC for 1 h and challenged with sanguinarine for 24 h. They were then harvested, and the indicated proteins were detected by Western blot analysis using anti-p-JNK, anti-JNK antibodies, and an ECL detection system. (C) The cells were treated with or without SP600125 for 1 h before challenge with sanguinarine for 24 h. The cells were analyzed using a flow cytometer to determine annexin-V. Data are reported as means ± SD of three independent experiments. For statistical analysis, the *t*-test was performed (*p<0.05 vs. untreated cells).

Many previous reports indicated that cytotoxic ROS signaling appeared to be mediated, in part, by activation of the c-Jun-N-terminal kinase (JNK) cascade rather than the p38 mitogen-activated protein kinase (MAPK) or the extracellular signal-regulated kinase (ERK) [34]–[36]. Thus, the current study investigated the involvement of JNK in sanguinarine-induced apoptosis. As shown in Fig. 6A, the phosphorylation of JNK was detectable after as little as 15–30 min of sanguinarine treatment and persisted for at least 1–4 h of the treatment. However, the ROS scavenger NAC completely blocked the enhanced phosphorylation of JNK (Fig. 6B). These results indicated that the JNK pathway was activated in a ROS-dependent manner in response to the presence of sanguinarine. To determine whether the activation of JNK participated in apoptosis, the effect of a specific JNK inhibitor, SP600125, on the sanguinarine-treated cells was tested. The results showed that the SP600125 pretreatment did not attenuate the accumulation of apoptotic cells relative to cells treated with SP600125 alone (Fig. 6C). The data indicate that ROS-dependent JNK phosphorylation does not occur upstream of sanguinarine-induced apoptosis in bladder cancer cells.

### Association of ROS-dependent Up-regulation of Egr-1 with Sanguinarine-induced Apoptosis

**Figure 7:**
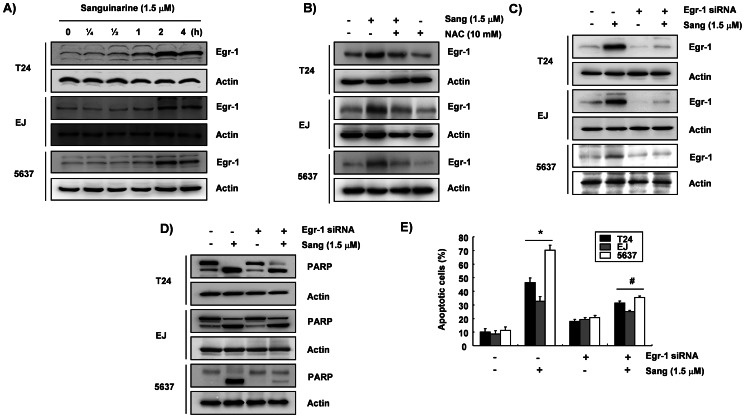
ROS-dependent induction of Egr-1 expression by sanguinarine in the bladder cancer cells. The cells were treated with sanguinarine for the indicated times (A) or treated with or without NAC for 1 h before challenge with sanguinarine for 2 h (B). Then, equal amounts of proteins (30 µg) were separated on SDS-polyacrylamide gels and transferred to nitrocellulose membranes. The membranes were probed with anti-Egr-1 antibody, and the proteins were visualized using an ECL detection system. (C–E) The cells were transfected with siRNA against human Egr-1 using a transfection reagent. After 24 h of transfection, the cells were treated with sanguinarine for another 2 h (C) or 24 h (D and E). They were then harvested, and the indicated proteins were detected by Western blot analysis (C and D). Furthermore, the cells were evaluated by a flow cytometer for annexin-V (D). The results are expressed as the mean ± SD of three independent experiments. The statistical significance of the results was analyzed with a Student’s *t*-test (*p<0.05 vs. untreated control; ^#^ *p*<0.01 vs. sanguinarine-treated cells).

Finally, the potential relationship between sanguinarine-induced apoptosis and Egr-1 expression was investigated. As shown in Fig. 7A, time-course analyses demonstrated that 1.5 µM of sanguinarine induced Egr-1 proteins within 2 h, and these did not return to baseline for 4 h. As sanguinarine generated ROS within 0.5 h, levels of ROS decreased after 2 h (Fig. 4), and the expression of Egr-1 by sanguinarine maximally increased over the 2–4 h treatment period, potential ROS-induced regulation of the induction of Egr-1 was investigated. Immunoblotting data indicated that blocking the generation of ROS by pretreatment of the cells with NAC markedly eliminated sanguinarine-induced Egr-1 proteins in the three cell lines (Fig. 7B). To investigate the role of Egr-1 in sanguinarine-induced apoptosis, Egr-1 gene expression was successfully down-regulated using Egr-1 siRNA (Fig. 7C). Its effect on PARP cleavage, as well as on apoptosis induction, was then evaluated. As shown in Fig. 7D and E, the inhibition of Egr-1 expression effectively mitigated the sanguinarine-induced degradation of PARP and the accumulation of apoptotic sub-G1 cells. The results confirm that the induction of apoptosis by sanguinarine occurs in an Egr-1-dependent manner and that an increase in ROS generation is required for activation of Egr-1 and the occurrence of sanguinarine-induced apoptosis in bladder cancer cells.

## Discussion

To study the mechanisms by which sanguinarine treatment induces apoptosis in bladder cancer cells, the present study examined a number of markers associated with apoptotic cell death. The mechanism of apoptosis is divided into two pathways: an extrinsic death receptor–mediated apoptotic pathway and an intrinsic mitochondria-mediated apoptotic pathway. Caspase activation is generally considered a key hallmark of apoptosis in these pathways. The extrinsic pathway is activated at the cell surface when a specific death ligand binds to its corresponding cell-surface receptor. In this pathway, caspase-8 acts as an initiator caspase, which activates the downstream effector caspases, such as caspase-3, -6, and -7. On the other hand, the intrinsic pathway has an apoptotic signal originating from within the cells, and it relies on the permeabilization of mitochondrial membranes to release apoptogenic mitochondrial proteins into the cytosol, thereby activating the initiator caspase-9. The activated caspase-9 initiates downstream events by directly cleaving and activating effector caspases, generating a fragment that activates the mitochondrial pathway [37], [38]. Apoptosis can also be regulated by several gene products, such as the Bcl-2 family of antiapoptotic and proapoptotic proteins, and the IAP family proteins, which are able to bind and inhibit caspases [39], [40]. In the mitochondrial death pathway, the ratio of expression of the proapoptotic proteins such as Bax and Bak and the antiapoptotic proteins such as Bcl-2 and Bcl-xL ultimately determines cell death or survival. In addition, caspase-8 mediates the intrinsic pathway via cleavage of the proapoptotic Bid protein, a BH3-only protein, to a truncated Bid (tBid) through translocation from the cytosol to the mitochondria, triggering mitochondrial dysfunction, followed by activation of caspase-9 [41]. This study demonstrated that the expression of the proapoptotic Bax showed an increase in sanguinarine-induced apoptosis, whereas the amount of antiapoptotic Bcl-2 and Bcl-xL remained relatively unchanged (Fig. 3A). The data also demonstrated that sanguinarine treatment induced apoptosis through activation of two initiator caspases, caspase-8 and -9, which were involved in the extrinsic and intrinsic pathways, respectively, as well as effector caspase-3 (Fig. 3B and C), which was associated with concomitant cleavage of PARP, an activated caspase-3 target substrate protein (Fig. 3B) [33]. Additionally, sanguinarine treatment reduced the expression of XIAP, a member of the IAP family of proteins (Fig. 3A), which have been reported to exert antiapoptotic effects because they function as direct inhibitors of activated caspases [37], [39]. Furthermore, exposure of cells to sanguinarine led to a significant reduction in the whole Bid, indicating that the proapoptotic protein, Bid, was truncated (Fig. 3A). Thus, the present data indicate that both extrinsic and intrinsic pathways may have contributed, at least in part, to the sanguinarine-induced apoptosis of the human bladder cancer cells.

Mounting evidence suggests that damaged mitochondria stimulate ROS generation and that disproportionate production of ROS induces apoptosis via the intrinsic pathway by causing damage to mitochondria through activation of caspases [17]–[19]. The data showed that sanguinarine treatment resulted in significantly increased ROS generation early in the process. Co-culture with NAC, a commonly used ROS scavenger, effectively blocked ROS generation (Fig. 4). In addition, blocking the generation of ROS completely prevented apoptosis (Fig. 5A) and recovered the sanguinarine-induced activation of caspases, the degradation of PARP, and the down-regulation of XIAP and whole Bid expression (Fig. 5B and C). These results indicated that ROS generation by sanguinarine is required for apoptosis induction in bladder cancer cells.

Recent studies have indicated that various apoptotic stimuli can rapidly activate MAPKs, which include JNK, ERK, and p38MAPK. Among them, the JNK pathway, as an upstream signaling pathway of caspase-3, may play an important role in triggering apoptosis in response to free radicals generated by ultraviolet radiation or direct application of H_2_O_2_ [42], [43]. Thus, the current study investigated whether this signal pathway was involved in the apoptotic effect of sanguinarine in bladder cancer cells. The data indicated that JNK phosphorylation occurs rapidly, within 30 min of sanguinarine treatment, and persists for at least 1–6 h after sanguinarine exposure (Fig. 6A). Pretreatment with NAC efficiently inhibited increased levels of JNK phosphorylation (Fig. 6B). However, SP600125, a specific inhibitor of JNK, did not attenuate the sanguinarine-induced apoptosis (Fig. 6C), suggesting that ROS-dependent JNK activation by sanguinarine cannot act as a mediator of the proapoptotic effects of sanguinarine in human bladder cancer cells.

Egr-1 is a member of the immediate-early gene family, and it can be rapidly induced by various stimuli [20]–[22], [44], [45].http://www.ncbi.nlm.nih.gov/pubmed/11331872 The Egr-1 protein plays a pivotal role in the regulation of cell growth, differentiation, and apoptosis. However, studies analyzing the functions of Egr-1 have yielded contradictory findings, with reports of both cytoprotective and proapoptotic functions in tumor cells. Although thttp://www.ncbi.nlm.nih.gov/pubmed/11948693he proapoptotic activity of Egr-1 may depend on the cell type and the nature of the stimulus, Egr-1-mediated apoptosis is associated with regulation of the expression of many tumor suppressor genes such as Egr-1 target genes [46]–[48]. In addition, the promoter site of Egr-1 contains the oxidative stress-responsive DNA sequences region [49], and most inducers of ROS-mediated signaling pathways increase the levels of Egr-1 [23]–[25], [27]. Therefore, the current study evaluated the involvement of Egr-1 in ROS-dependent apoptosis of bladder cancer cells by sanguinarine. The data indicated that sanguinarine markedly increased the levels of Egr-1 proteins after 2 h. Blocking the generation of ROS with NAC diminished this effect (Fig. 7A and B). Furthermore, the inhibition of Egr-1 expression by siRNA-mediated knockdown significantly decreased the apoptosis by sanguinarine (Fig. 7C and E). Although the reduction in PARP degradation was only partial (Fig. 7D), the results indicated that Egr-1 plays an important role as a gene regulator in the apoptosis of bladder cancer cells treated with sanguinarine.

In conclusion, the present data indicate that human bladder cancer cells undergo apoptosis in response to treatment with sanguinarine and that this occurs through a mitochondria-mediated pathway, which requires ROS generation upstream and the subsequent activation of caspases. The results of this study also suggested that Egr-1, as a target gene of ROS, plays an important role in the regulation of sanguinarine-induced apoptosis. The current data may provide increased understanding of the mechanisms underlying the anticancer activity of sanguinarine, and further dissection of the mechanisms may lead to the development of therapeutic approaches for the attenuation of bladder cancer.
